# Examining the Mental Workload Associated With Digital Health Technologies in Health Care: Protocol for a Systematic Review Focusing on Assessment Methods

**DOI:** 10.2196/29126

**Published:** 2021-08-03

**Authors:** Lisanne Kremer, Myriam Lipprandt, Rainer Röhrig, Bernhard Breil

**Affiliations:** 1 Faculty of Health Care Niederrhein University of Applied Sciences Krefeld Germany; 2 Institute of Medical Informatics RWTH Aachen University Aachen Germany

**Keywords:** mental workload, cognitive load, assessment, healthcare workers, health information system, digital health technology, health care professionals, stress, eyetracking

## Abstract

**Background:**

The workload in health care is high; physicians and nurses report high stress levels due to a demanding environment where they often have to perform multiple tasks simultaneously. As a result, mental health issues among health care professionals (HCPs) are on the rise and the prevalence of errors in their daily tasks could increase. Processes of demographic change are partly responsible for even higher stress levels among HCPs. The digitization of patient care is intended to counteract these processes. However, it remains unclear whether these health information systems (HIS) and digital health technologies (DHT) support the HCPs and relieve stress, or if they represent a further burden. The mental construct that describes this burden of technologies is mental workload (MWL). Work in the clinic can be viewed as working in safety-critical environments. Particularly in this sensitive setting, the measurement methods of MWL are relevant, mainly due to their strongly differing levels of intrusiveness and sensitivity. The method of eye tracking could be a useful way to measure MWL directly in the field.

**Objective:**

The systematic review aims to address the following questions: (1) In which manner do DHT contribute to the overall MWL of HCPs? (2) Can we observe a direct or indirect effect of DHT on MWL? (3) Which aspects or factors of DHT contribute to an increase in MWL? (4) Which methods/assessments are applied to measure MWL related to HIS/DHT? (5) What role does eye tracking/pupillometry play in the context of measuring MWL? (6) Which outcomes are being assessed via eye tracking?

**Methods:**

Following the PRISMA (Preferred Reporting Items for Systematic Review and Meta-Analysis) statement, we will conduct a systematic review. Based on the research questions, we define keywords that we then combine in search terms. The review follows the following steps: literature search, article selection, data extraction, risk of bias assessment, data analysis, and data synthesis.

**Results:**

We expect results as well as a finalization of the review in the summer of 2021.

**Conclusions:**

This review will evaluate the impact of DHT on the MWL of HCPs. In addition, assessment methods of MWL in the context of digital technologies will be systematically analyzed.

**Trial Registration:**

PROSPERO (International Prospective Register of Systematic Reviews) CRD42021233271; https://www.crd.york.ac.uk/PROSPERO/display_record.php?ID=CRD42021233271

**International Registered Report Identifier (IRRID):**

DERR1-10.2196/29126

## Introduction

### Background

The workload for health care workers has remained high for many years [1,2]. Several factors contribute to this trend and result in different effects for employees and the health care system [3]. Factors that promote a high workload include understaffing, long working hours [4], and information overload [5]. Work-related stress has become one of the main challenges in the health care sector [6] and has different impacts on employees. Nurses in particular report high levels of work-related stress that can lead to negative physical and psychological effects for them as well as for their patients [7]. Nurses describe themselves as feeling empty and report depressive symptoms [8,9]. In Germany, health care professionals (HCPs) have an above-average number of sick days compared to workers in other sectors; overall, there was a 29% increase in sick days between 2004 and 2018 [10]. In addition to musculoskeletal disease diagnoses, which account for the majority of sick leaves, absences due to mental illness are increasing significantly [11].

Partly responsible for the workload-promoting factors described above are the consequences of demographic changes that have led to an increase in the number of multimorbid older adult patients and a decline in the number of nursing staff. The transformation process of digitization in health care is a chance to counteract this change and its consequences. However, in Germany in particular, the process is proceeding very slowly; Germany is ranked 19th of 27 countries in Bertelsmann’s Digital Health Index [12]. The application of digital health technology (DHT) is an important factor of this digitization process. DHT in the context of this work means technologies that are directly linked to outpatient and inpatient care and are applied by nurses or physicians. DHT includes hospital information systems (HIS), medical devices, and other digital applications that support patient care from the perspective of HCPs.

In addition to the positive effects of the use of DHT, there is also evidence to suggest that the use of DHT causes an extra load. This may be due to a lack of usability and user involvement as well as poor implementation processes [13,14].

Poor usability and other factors rooted in technologies can cause a high mental workload (MWL) [14]. High workloads can result in a more error-prone performance—even for experts—induced by difficulties in decision-making processes [15].

Working with patients can be considered a safety-critical environment. This means that many tasks, varying in complexity, occur within limited time windows.

In this context, decisions must be made all the time and are supported by different systems (eg, HIS) through the structured and standardized presentation of information. The interaction between users and systems is complex and interdependent, which makes it difficult to predict the effects of the systems on the users [16].

High workload or overload caused by several factors (including technology) can have a severe impact. Aside from the negative impact on patient care due to a potential increase in errors, overload can also have a negative impact on the health of HCPs, potentially resulting in technostress, mental health issues (eg, depression, burnout), and decreased job satisfaction. These are only a few of the potential negative effects of overload [17]. There is growing evidence that DHT are contributing to increasing mental health problems (eg, burnout) among health care workers [18,19].

In order to identify possible causes of mental health problems in physicians and nurses (eg, emerging burnout [20]), the investigation of MWL in different situations is a possible approach.

### Mental Workload

MWL can be defined using different approaches and is usually influenced by different and multiple factors. It is multidimensional, multifaceted, and one of the most important variables to understand and predict human performance.

The possible definitional approaches of workload can be derived from two different perspectives: (1) MWL as an external variable referring to task requirements (ie, the amount of work and the number of tasks to be completed in a limited time [task load]) and (2) interaction between task and human resources resulting in a subjective psychological experience [21,22].

Summarizing different approaches, we can define MWL as the amount of attentional resources that are required to perform a task mediated by task demands and experience [15,23,24]. Following this definition, the state of overload is reached when the task demands are too high while the user's resources are limited. In contrast to this is the condition of underload, which occurs when the task requirements are too low while resources are sufficient. In both cases, the result is poorer performance [25]. Mental states such as a high workload or underload play a critical role in the occurrence of errors as well as preventable adverse events [26]. Regardless of how competent and/or experienced an HCP is, this type of mental state can lead to a higher frequency of errors.

### Assessment of Mental Workload

MWL assessments were first developed and applied in other safety-critical environments such as aviation/aerospace and nuclear power plants. Safety-critical environments have similar conditions (already described). Due to these similar conditions, workload assessment could also be a useful approach in the clinical setting.

MWL can be assessed using different techniques. A distinction between analytical and empirical methods may be drawn. Analytical methods tend to be used in system development, while empirical methods are employed when workload is to be measured directly in the executing system or in the simulation [21].

Analytical assessment methods are simulation models, expert opinions, or task analyses. Empirical methods are distinguished into three different categories: performance measures, subjective methods, and physiological techniques [15]. Performance measures refer to the measures of the primary and secondary task.

Depending on the situation and the underlying question, one or more of these techniques are appropriate to apply. Several factors should be considered when selecting assessments, including sensitivity, diagnostic accuracy, intrusiveness, validity, reliability, simplicity of use, and user acceptance [27].

### Objectives

DHT may contribute to the heavy workload in health care. MWL can best reflect the workload caused by technology. In addition to the existence of some methodological issues (eg, assessing MWL in the field), there are also some knowledge gaps concerning MWL caused by DHT.

The planned systematic review intends to identify the impact of DHT, particularly HIS, on the MWL of health care workers. In addition, the review will aim to assess what methods are currently being used in health care to measure MWL relating to DHT. In particular, the application of eye tracking or pupillometry as an assessment method will be investigated.

### Research Questions

The review will seek to answer the following research questions:

- 1. In which manner do DHT contribute to the overall MWL of health care workers?
  - 1.1. Can we observe a direct or indirect effect of DHT on MWL?
  - 1.2. Which aspects or factors of DHT contribute to an increase in MWL?
- 2. Which methods/assessments are applied to measure MWL related to HIS/DHT?
  - 2.1. What role does eye tracking/pupillometry play in the context of measuring MWL?
  - 2.2. Which outcomes are being assessed via eye tracking?

## Methods

### Study Registration

The protocol is registered in the International Prospective Register of Systematic Reviews (PROSPERO; CRD42021233271). This protocol follows the PRISMA-P (Preferred Reporting Items for Systematic Review and Meta-Analysis Protocols) 2015 guidelines [28].

### Eligibility Criteria

We define the inclusion criteria for this systematic review according to the PICO framework [29] and the research questions. Inclusion criteria relate to the study population (P), intervention (I), outcome (O) of the study, and study setting (C). In addition to these criteria, we include studies by study design as detailed below.

### Study Design

All types of study designs reporting original primary data as well as systematic reviews that align with our other inclusion criteria will be included. We will exclude commentaries, letters, guidelines, and narrative reviews.

### Study Participants

We focus on HCPs who work with HIS or DHT and who are directly engaged in patient care. These can be nurses, physicians, radiology assistants, or other clinicians. It is essential that the participants are supported by the HIS/DHT in their daily work with patients. We exclude studies that focus on patients who use digital technologies.

### Intervention

We include studies that investigate the effects that HIS/DHT have on workers’ MWL. The focus lies on the evaluation of whether there is a direct or indirect effect of DHT on workers' MWL. Since the second research question concerns the extent to which eye tracking is commonly used as a measurement method, we focus on the inclusion of studies that apply eye tracking. We exclude studies that investigate related constructs such as technostress.

### Study Setting

We include all studies that take place in inpatient or outpatient care. We exclude studies that focus on the measurement of MWL in other contexts (eg, aviation).

### Information Sources

The following databases were systematically searched between February 28 and March 15, 2021, using defined keywords (and synonyms) like “mental workload,” “health information system,” “assessment,” “health care professionals,” and “eye tracking” that resulted in specified search strings: MEDLINE (PubMed), Web of Science, Academic Search Premier and CINAHL (both EBSCO), and PsycINFO. Additionally, we will search for relevant research in the reference sections of included studies as well as those of relevant recently published reviews. Following PRISMA-P [28], we organized the search terms by database and question in a separate document (Multimedia Appendix 1).

### Search Strategy

The search strategy includes four categories, each represented by keywords and synonyms: technologies used (eg, HIS), population (eg, health care professionals), methods (eg, assessment), and MWL. In addition, eye tracking will be added for questions 2.1 and 2.2. The terms are linked by the Boolean operators AND or OR.

We restrict our search to articles published in the period between 2000 and 2021. This search time frame was chosen because it documents the development of the current generation of prehospital communication technology, such as telemedicine and electronic patient care reports [30]. The literature search is limited to articles written in English or German since both reviewers have a sufficiently high level of fluency in these languages.

### Study Records

#### Data Management

Citavi is used for literature handling (ie, import and further screening). The Rayaan web-based screening tool is used to perform abstract screening and full-text analysis in a structured way. In this context, the inclusion and exclusion criteria are also provided; they will be the basis for the abovementioned analysis process. The included articles will be then imported to a Microsoft Excel (Microsoft Corp) spreadsheet.

#### Selection Process

The selection process will be performed by two reviewers (LK and BB; if a consensus cannot be reached, ML and RR will serve as additional reviewers) according to the PRISMA guidelines and will be displayed in a flowchart. First, both reviewers will assess the studies regarding the inclusion and exclusion criteria for abstract screening. In the next step, the full texts of the resulting studies will again be assessed independently. Finally, we will search the references of the papers for further potentially eligible studies. In case of disagreements in any of the phases, a discussion between the two reviewers (LK and BB) based on the inclusion criteria will be attempted first. If the discussion is inconclusive, a third reviewer (ML or RR) will be involved.

#### Data Collection Process

For data extraction, an Excel spreadsheet based on the outcomes of the review will be used. To ensure uniformity across reviewers, we will conduct a pretest standardization exercise before starting the data extraction process. Each reviewer will extract the themes of interest to an Excel spreadsheet. The extracted data items are presented below.

#### Data Items

LL and BB will read the full texts and extract information concerning identified and relevant aspects of the studies. We will differentiate between main study characteristics, measurements and outcomes, and relevant findings and recommendations. The aspects are aggregated in Table 1, Table 2, and Table 3.

**Table 1:** Systematic analyses of the main study characteristics.

Theme	Indicator
Objectives	Aims
Assessments	Eg, questionnaires
Quality criteria of applied assessments	Reported/not reported Type of quality criterium (eg, internal consistency)
Outcomes	Mental workload related to digital health technologies Factors of digital health technologies contributing to mental workload Assessment type Role of eye tracking
Type of digital health technology	Eg, apps, health information systems

**Table 2:** Systematic analyses of measurements and outcomes (study characteristics).

Theme	Indicator
Study identification	Author Reference number
Setting of target	Eg, hospital, outpatient setting
Study design	Cross-sectional, longitudinal Quantitative, qualitative, mixed methods
Sample characteristics	Sample size Age Sex
Population type	Eg, physicians, nurses

**Table 3:** Systematic analyses of the main findings.

Theme	Indicator
Overall workload level	Assessed/not assessed High, medium, low
Mental workload related to digital health technologies	High, medium, low
Factors of digital health technologies contributing to mental workload	Eg, lack of error tolerance
Eye tracking	Applied Field of application Study settings
Outcomes measured by eye tracking	Qualitative (eg, heat map) Quantitative (eg, fixation duration) Mental workload assessment

In addition to the descriptive presentation of study characteristics and findings, we are aiming to extract factors or aspects of DHT that contribute to an increasing MWL. Furthermore, we would like to extract how the included studies assess workload and in which settings eye tracking is used with regard to specific outcomes. Based on the extraction, we would like to develop an overview of the methods that can be used to measure MWL caused by DHT and provide meaningful and valid data.

**Figure 1:**
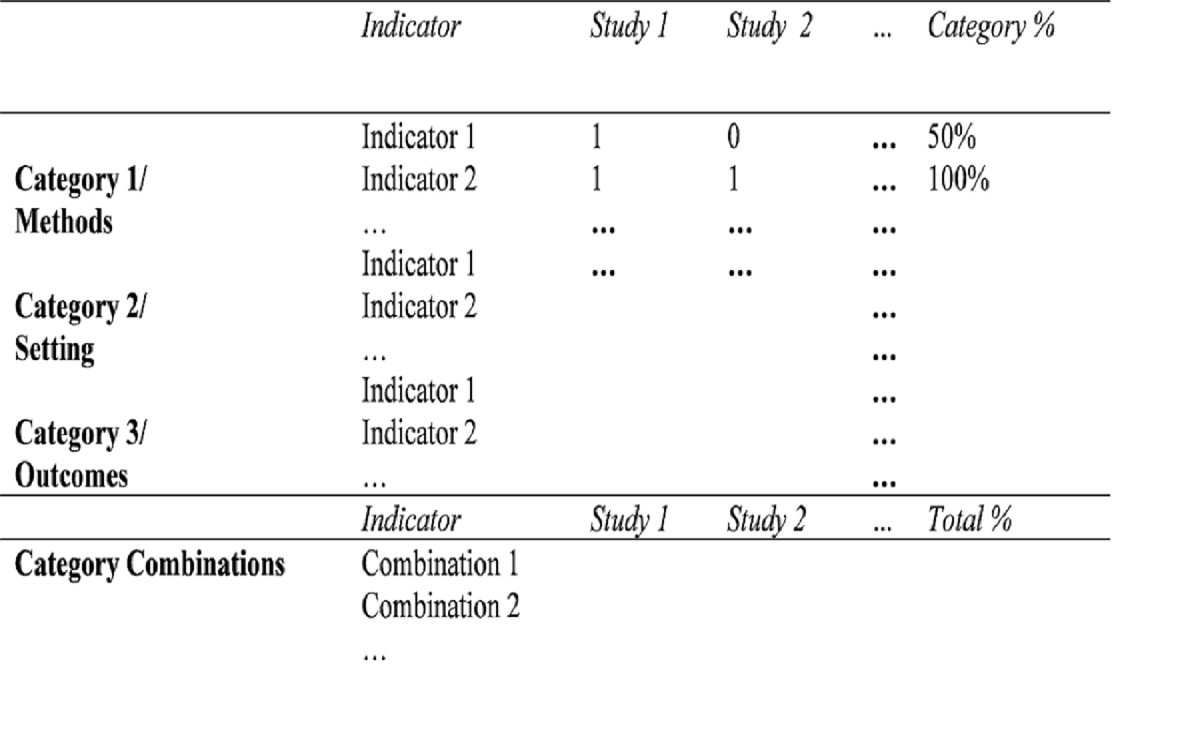
Example of systematic tabulation of methods, setting, outcomes, and combined investigation procedures.

The methods, settings, and outcomes will be organized into logical categories that are rated by the reviewers. The typical categories of methods referring to MWL assessments are analytical or empirical techniques. Typical categories for settings are laboratory or field. Categories referring to assessed outcomes have to be defined during the reviewing process. In each category, we will extract how often an indicator for a category was applied (category percentage, ie, method applied/n studies) and how often combinations of specific indicators were used (total percentage, eg, method A with setting B and outcome C; combination applied/N studies). A typical indicator for the category empirical technique would be a questionnaire. If an indicator was identified, the reviewers fill the row with a 1; if no indicator was identified (eg, if the method was not applied), the table is filled with a 0. An example is displayed in Figure 1.

### Outcomes

The primary outcome of the first research question is to explore the correlations between DHT and the MWL of HCPs. The secondary outcome is to investigate the type of effect (direct/indirect) DHT has on the MWL of HCPs as well as the aspects of DHT that contribute to MWL.

The primary outcome of the second research question is the exploration of the best method to determine this relationship. Particular attention will be given to the role of eye tracking technology, which will be included as a secondary outcome.

### Risk of Bias in Individual Studies

For the review, two authors will independently rate the methodological quality of the identified studies using the Joanna Briggs Institute Critical Appraisal Tool [31]. An initial screening of studies that could be included indicates a small proportion of studies with an experimental design and adequately defined criteria for conducting the study and analyzing the data. Disagreements will be resolved via discussion (LK and BB) or by a third reviewer (ML or RR), if necessary.

### Data Analysis and Synthesis

After screening the search results, we do not expect to be able to conduct a meta-analysis. A first look revealed that comparing the study designs and effect measures of studies will be difficult. This may be explained by the explorative character of the review and the potentially low level of evidence, especially regarding eye tracking. Instead, we will perform a descriptive analysis to summarize the data, starting with a comparison of evaluation methods (qualitative, quantitative, or mixed methods) and survey methods. To do this, we will first compare the studies in terms of the evaluation methods used (qualitative, quantitative, mixed methods), followed by a comparison of survey methods.

For data synthesis, we use two nonquantitative approaches: tabulation and a narrative approach. Table 1 and Table 2 describe the tabular synthesis of potential findings.

In a first step, all main characteristics of each study will be extracted (ie, study design, setting of target population, sample size, age, sex, population type). Studies that do not report those main characteristics and those with a sample size under 20 participants will be excluded. We will analyze studies regarding objectives, outcomes, and assessments, as well as type of DHT. Data on overall MWL in studies, MWL levels related to DHT, quality criteria of assessments, applied eye tracking, and outcomes assessed via eye tracking will be extracted.

All included studies are evaluated with regard to their risk of bias. A textual narrative synthesis of all included studies will be made and the comparable findings will be synthesized. Additionally, a descriptive analysis of eye tracking measures is planned.

## Results

As the systematic review is currently ongoing, no results are available as of yet. The preliminary searches have been completed and the piloting of the study selection process as well as the formal screening against eligibility criteria has started. We are currently analyzing the data and expect to complete the review in summer 2021.

## Discussion

The aim of the review is to show which methods are currently used to measure MWL in health care and the impact of such technologies on the workload of HCPs. Additionally, the role of eye tracking should be evaluated.

In the discussion section of the review, we will discuss the results and the methodological quality of the findings, strengths and weaknesses of the review (limitations), and research gaps and opportunities for future research.

## Appendix

Multimedia Appendix 1 Search Strings: Performed searches sorted by question and database.

## Abbreviations

DHT: digital health technology
HCP: health care professional
HIS: health information system
MWL: mental workload
PRISMA-P: Preferred Reporting Items for Systematic Review and Meta-Analysis Protocols
