# Supplementary material for: Examining the Mental Workload Associated With Digital Health Technologies in Health Care: Protocol for a Systematic Review Focusing on Assessment Methods
Source: JMIR Res Protoc. 2021 Aug 3;10(8):e29126. doi: 10.2196/29126 (PMC8371485; doi:10.2196/29126)
Supplement: Multimedia Appendix 1 [file resprot_v10i8e29126_app1.docx]

**Search Strategy Documentation**

| **Information Sources** | **Medline, Web of Science, Academic Search Premier/ CINAHL (EBSCO), PsycInfo** |
| --- | --- |
|  |  |

| **Source:** | **Date of search** | **Search strategy used (keywords & Boolean)** | **Search Limits or filters (e.g. dates, language)** | **# results found** |
| --- | --- | --- | --- | --- |
| Medline  Q1 | 10.03.2021 | ((((((applications, medical informatics[MeSH Terms]) OR (record, electronic health[MeSH Terms])) OR (record, electronic medical[MeSH Terms])) OR (health information technology[MeSH Terms])) OR (design, medical device[MeSH Terms])) AND **Search** (((((employee workload[MeSH Terms]) OR (stress, psychological[MeSH Terms])) OR ("mental workload")) OR ("cognitive load"))) AND (((((health personnel[MeSH Terms]) OR (administrator, nurse[MeSH Terms])) OR (aide, nurses[MeSH Terms])) OR (assistant, physicians[MeSH Terms])) OR (physicians[MeSH Terms])) | 2000-2021  English or German | 373 |
| Medline  Q2 | 10.03.2021 | ((((((applications, medical informatics[MeSH Terms]) OR (record, electronic health[MeSH Terms])) OR (record, electronic medical[MeSH Terms])) OR (health information technology[MeSH Terms])) OR (design, medical device[MeSH Terms])) AND (((((employee workload[MeSH Terms]) OR (stress, psychological[MeSH Terms])) OR ("mental workload")) OR ("cognitive load"))) AND ((((((assessement) OR (questionnaire)) OR (survey)) OR (scale)) OR (test)) | 2000-2021  English or German | 1209 |
| Medline  Q3 | 10.03.2021 | ((((((employee workload[MeSH Terms]) OR (stress, psychological[MeSH Terms])) OR ("mental workload")) OR ("cognitive load")) AND (((((eye movement[MeSH Terms]) OR (pupillometry)) OR ("eye tracking")) OR ("eye tracker")) OR ("eye movement measurement")) AND (english[Filter] OR german[Filter])) AND (Health Care) | 2000-2021  English or German | 21 |
| Academic Search Premier/ CINAHL  (EBSCO)  Q1 | 10.03.2021 | ((electronic+health+records)+OR+(electronic+medical+record)+OR+(health+information+systems)+OR+(digital+health+technology)+OR+(electronic+health+record+system))+AND+((workload)+OR+(mental+workload)+OR+(cognitive+workload)+OR+(cognitive+load)+OR+(information+overload+AND+stress))+AND+((health+personnel)+OR+(health+care+workers)+OR+(health+care+professionals)+OR+(doctors+OR+physicians)+OR+(nurse)) | 2000-2021  English or German | 633 |
| Academic Search Premier/ CINAHL  (EBSCO)  Q2 | 10.03.2021 | ((electronic+health+records)+OR+(electronic+medical+record)+OR+(health+information+systems)+OR+(digital+health+technology)+OR+(electronic+health+record+system))+AND+((workload)+OR+(mental+workload)+OR+(cognitive+workload)+OR+(cognitive+load)+OR+(information+overload+AND+stress))+AND+((health+personnel)+OR+(health+care+workers)+OR+(health+care+professionals)+OR+(doctors+OR+physicians)+OR+(nurse))+AND+((measurement+tool)+OR+(scale)+OR+(test)+OR+(assessment)+OR+(questionnaire+OR+survey)+OR+(methods)) | 2000-2021  English or German | 460 |
| Academic Search Premier/ CINAHL  (EBSCO)  Q3 | 10.03.2021 | ((workload)+OR+(mental+workload)+OR+(cognitive+workload)+OR+(cognitive+load)+OR+(information+overload+AND+stress))+AND+((health+personnel)+OR+(health+care+workers)+OR+(health+care+professionals)+OR+(doctors+OR+physicians)+OR+(nurse))+AND+((eye+tracking+OR+eye+tracker+OR+eye+movement+measurements+OR+visual+tracking)+OR+(fixation)+AND+(pupillometry)) | 2000-2021  English or German | 16 |
| Web of Science  Q1 | 10.03.2021 | TS=(Health Record* OR Electronic Health Record* OR Health Information System* OR Health Technology*) AND TS=(cognitive load OR workload OR mental workload OR information overload OR stress OR Human Channel capacity)  AND  TS=(cognitive load OR workload OR mental workload OR information overload OR stress OR Human Channel capacity) | 2000-2021  English or German | 1971 |
| Web of Science  Q2 | 10.03.2021 | TS=(Health Record* OR Electronic Health Record* OR Health Information System* OR Health Technology*) AND TS=(cognitive load OR workload OR mental workload OR information overload OR stress OR Human Channel capacity)  AND  TS=(cognitive load OR workload OR mental workload OR information overload OR stress OR Human Channel capacity)  AND  (TS=(measurement method* OR measurement OR assessment OR test OR scale OR questionnaire OR survey OR instrument*) NOT TS=(Screening) NOT TS=(diagnosis)) | 2000-2021  English or German | 845 |
| Web of Science  Q3 | 10.03.2021 | TS=(Health Record* OR Electronic Health Record* OR Health Information System* OR Health Technology*)  TS=(cognitive load OR workload OR mental workload OR information overload OR stress OR Human Channel capacity)  TS=(eye movement* OR eye-tracking* OR saccade* OR fixation* OR psychophysiology OR eyemovements OR eyetracking) |  | 115 |
| PsycInfo | 08.03.2021 | (“Health Record*” OR “Electronic Health Record*” OR “Health Information System*” OR “Health Technology*”)  AND  (“cognitive load” OR workload OR “mental workload” OR “information overload” OR “Human Channel Capacity”)  AND  (“health personnel” OR physician* OR nurse* OR doctor*)  OR  (“Health Record*” OR “Electronic Health Record*” OR “Health Information System*” OR “Health Technology*”)  AND  (“cognitive load” OR workload OR “mental workload” OR “information overload” OR “Human Channel Capacity”)  AND  (eyemovement* OR “eye-tracking*” OR saccade* OR fixation* OR eye tracking OR saccade* OR fixation*) | 2000-2021  English or German | 1882 |

| **All databases** |  |  |  | **7525** |
| --- | --- | --- | --- | --- |
|  |  |  | *Duplicates* | *1965* |
|  |  |  | Search results | **5563** |

(*adapted from The University of Notre Dame, Australia;* [*https://library.nd.edu.au/researchers/systematicreviews/process*](https://library.nd.edu.au/researchers/systematicreviews/process)*; [accessed: 05-05-2021)*
